# Colorectal cancer in ulcerative colitis: unraveling mechanisms and advancing surveillance and chemoprevention

**DOI:** 10.3389/fonc.2026.1793470

**Published:** 2026-04-15

**Authors:** Haiyan Lu, Xiangdang Hu, Yan Chen, Jie He

**Affiliations:** 1 Anorectal surgery, The Second Affiliated Hospital of Hunan University of Chinese Medicine, Changsha, China; 2 College of Traditional Chinese Medicine, Changsha Medical University, Changsha, China; 3 The Third Hospital of Changsha, Changsha, China

**Keywords:** chemoprevention, colitis-associated cancer, colorectal cancer, surveillance colonoscopy, ulcerative colitis

## Abstract

Patients with ulcerative colitis (UC) have an increased risk of developing colorectal cancer through inflammation-driven carcinogenesis distinct from sporadic disease. Although the relative risk remains elevated, contemporary population-based studies indicate a substantial decline in absolute incidence, likely reflecting improved disease control, biologic therapies and structured surveillance colonoscopy. This review synthesizes current evidence on the epidemiology and determinants of colitis-associated colorectal cancer (CAC), highlighting key risk modifiers such as disease extent and duration, persistent inflammation, family history, and primary sclerosing cholangitis. We further summarize advances in understanding the molecular and immunologic mechanisms underlying CAC, including genomic instability, immune dysregulation, oxidative stress, microbiome alterations, and tumor microenvironment remodeling. Emerging molecular and histologic biomarkers that may enhance risk stratification and guide precision surveillance are discussed. In addition, contemporary surveillance approaches and evolving chemoprevention strategies are critically evaluated. Collectively, this review outlines current challenges and future directions for individualized CRC prevention in patients with UC.

## 1 Introduction

Ulcerative colitis (UC) is a chronic, relapsing inflammatory bowel disease (IBD) characterized by continuous mucosal inflammation of the colon, beginning in the rectum and extending proximally to a variable extent (1). Beyond its substantial impact on health, UC is associated with an increased risk of colorectal cancer (CRC), a complication that has long represented one of the most feared long-term outcomes of the disease (2). Colitis-associated colorectal cancer (CAC) differs biologically, clinically, and epidemiologically from sporadic CRC, arising in the context of chronic inflammation and following a distinct carcinogenic pathway. Consequently, prevention and early detection of CRC have become central objectives in the long-term management of UC (3). The high cumulative CRC risks in UC, approaching 18% after 30 years of disease duration (4). The earlier diagnosis, improved disease control, widespread use of immunomodulators and biologic therapies and the implementation of structured surveillance colonoscopy programs have contributed to a substantial decline in the absolute risk of CRC among patients with UC (5). Despite these improvements, the risk of CRC in UC remains higher than in the general population, underscoring the continued need for effective prevention and surveillance strategies (6).

The relationship between UC and CRC is complex and heterogeneous. Cancer risk is not uniform across all patients but is influenced by multiple disease-related, host-related and environmental factors (7). Disease extent and duration remain among the most important determinants with extensive and long-standing colitis conferring the highest risk. Coexisting primary sclerosing cholangitis (PSC) represents a particularly high-risk phenotype and is often associated with earlier onset, right-sided predominance and more aggressive neoplasia (8). Additional modifiers include age at UC diagnosis, family history of CRC, severity and persistence of inflammation and possibly sex and lifestyle factors (8). Recognition of this heterogeneity has led to a shift away from uniform surveillance approaches toward individualized risk stratification and tailored monitoring strategies (9).

At the mechanistic level, CAC develops through an inflammation-dysplasia-carcinoma sequence that differs from the classical adenoma-carcinoma pathway observed in sporadic CRC (10). Chronic mucosal inflammation promotes repeated cycles of epithelial injury and regeneration, contributing to genomic instability, epigenetic alterations and field cancerization across extensive segments of the colon (11). Dysplasia in UC is frequently flat, multifocal and endoscopically subtle which presents considerable challenges for detection during routine endoscopic evaluation (11, 12). At the molecular level, early alterations in tumor suppressor genes such as TP53 along with immune dysregulation, oxidative stress, microbiota dysbiosis and remodeling of the tumor microenvironment contribute to accelerated carcinogenesis (12). Improved understanding of these mechanisms is essential for developing more effective surveillance strategies and identifying potential biomarkers and therapeutic targets (13).

Surveillance colonoscopy remains the cornerstone of CRC prevention in UC. Advances in endoscopic imaging technologies, particularly high-definition colonoscopy and dye-based or virtual chromoendoscopy have significantly improved dysplasia detection rates (13). Current international guidelines increasingly recommend risk-adapted surveillance intervals based on individual patient characteristics rather than fixed schedules (13). Nevertheless, several challenges remain in real-world clinical practice including variability in access to specialized expertise, inconsistent adherence to surveillance recommendations, interobserver variability in dysplasia interpretation, and uncertainty regarding the optimal management of detected lesions (14).

In parallel, increasing attention has been directed toward chemoprevention as an adjunct to surveillance (13). Several conventional UC therapies, particularly 5-aminosalicylates, immunomodulators and biologics may reduce CRC risk indirectly by maintaining sustained control of intestinal inflammation (15). Other potential chemopreventive agents including statins, ursodeoxycholic acid and dietary or microbiota-targeted interventions are also being investigated, although current evidence remains heterogeneous (16). Balancing potential benefits with safety considerations, cost and long-term adherence remains an important challenge in clinical practice (16).

This review provides a comprehensive and contemporary overview of colorectal cancer in patients with UC. The authors summarize current evidence on epidemiology and risk stratification, examine the molecular and immunologic mechanisms underlying CAC and evaluate emerging biomarkers that may improve early detection and risk prediction. In addition, the review critically discusses modern surveillance approaches and evolving chemoprevention strategies.

## 2 Epidemiology and risk stratification

**Figure 1:**
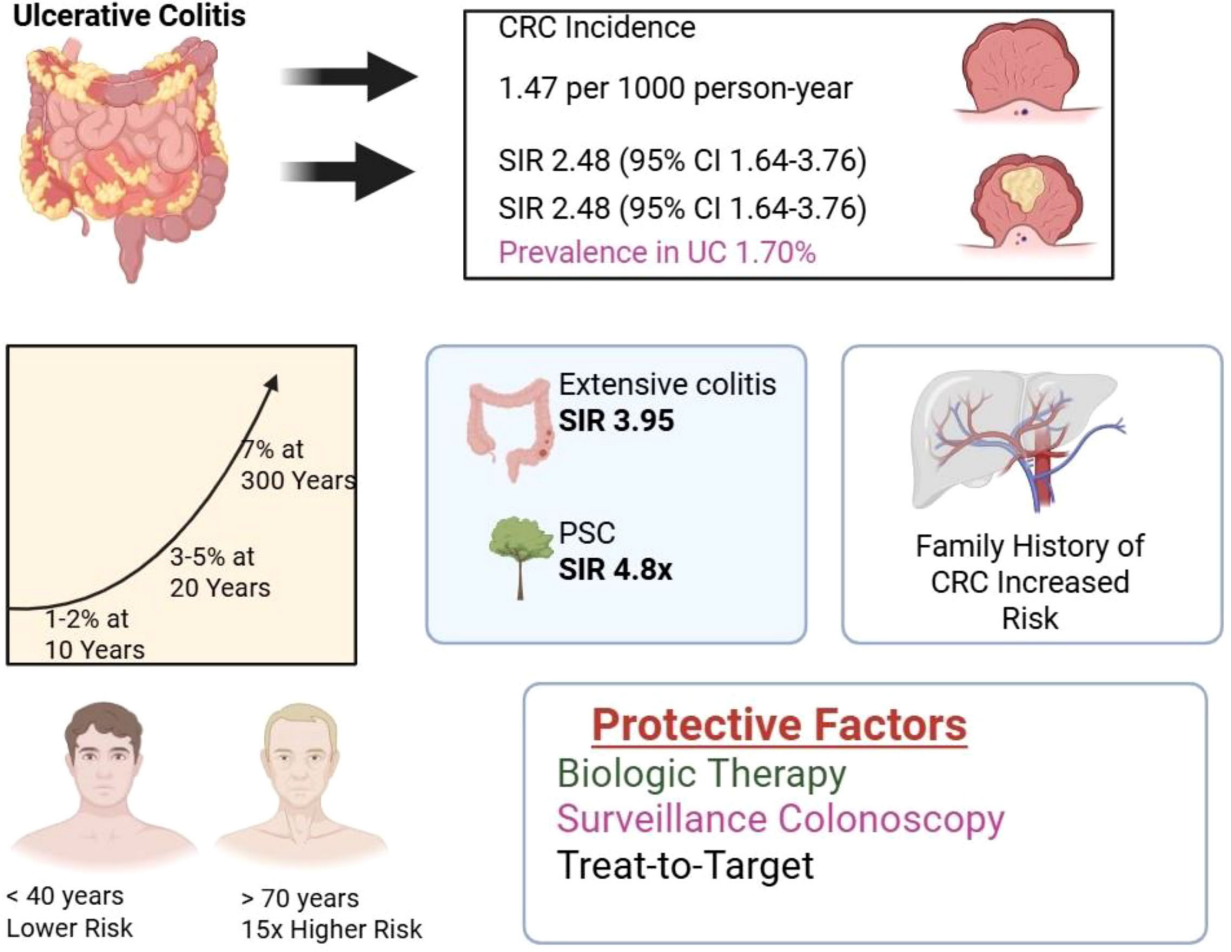
Epidemiology and risk stratification in UC and CRC.

Ulcerative colitis (UC) elevates colorectal cancer (CRC) risk compared to the general population, though absolute risks have declined in the biologic’s era. Recent meta-analyses report a pooled CRC incidence rate is1.47 cases per 1000 person-years, standardized incidence ratio (SIR) is 2.48 per 1000 person-years and prevalence of 1.70% among UC patients (17). Cumulative risks remain low: approximately 1-2% at 10 years, 3-5% at 20 years, and 7% at 30 years post-diagnosis, far below historical estimates of 18% at 30 years (5, 17) (**Figure 1**).

This decline reflects widespread maintenance therapies, surveillance colonoscopy and treat-to-target strategies with SIRs dropping to 1.0-2.75 in recent cohorts. Compared with left-sided colitis, extensive colitis is associated with an approximately threefold higher risk of CAC (SIR 3.95, 95% CI 2.56–6.09), while PSC confers a 4.8-fold increase in risk. Family history of CRC independently increases susceptibility, irrespective of IBD extent. Age at UC diagnosis strongly predicts outcomes: patients over 70 years face 15-fold higher CRC hazard than those under 40. Sex differences are minimal (SIR ~2.1-2.2 for both), though some data suggest slightly higher male risk (4, 18–20).

## 3 Pathogenesis: from chronic inflammation to carcinoma

Chronic inflammation in UC fuels CAC via a unique inflammation dysplasia carcinoma sequence bypassing the adenoma precursor typical of sporadic CRC. Repeated mucosal injury promotes field cancerization, genetic/epigenetic instability, immune dysregulation and microenvironmental remodeling which accelerate transformation (21, 22).

### 3.1 Inflammation-dysplasia-carcinoma sequence

Chronic inflammation in UC establishes the CAC pathway, fundamentally differing from sporadic CRC’s adenoma-carcinoma sequence. Sporadic CRC begins with APC-mutated adenomas progressing slowly via chromosomal instability or microsatellite instability; CAC skips adenomas, following a rapid inflammation-dysplasia-carcinoma model driven by mucosal injury (10, 21).

Repeated cycles of epithelial damage and repair create “field cancerization,” where broad inflamed regions accumulate mutations, fostering multifocal, flat dysplasia. Aneuploidy appears early in non-dysplastic mucosa, predicting dysplasia risk, with clonal expansion under inflammatory pressure. Progression advances from indefinite dysplasia (reactive changes) to low-grade dysplasia (LGD), high-grade dysplasia (HGD), and invasive carcinoma. LGD confers ~4-15% 5-year CRC risk; HGD nears 50% (10, 22, 23).

Key drivers include reactive oxygen/nitrogen species causing DNA adducts, defective repair, and p53 stabilization early (50% in dysplasia vs. late in sporadic CRC). Inflammation sustains proliferation via cytokines, selects apoptosis-resistant clones, and impairs immune surveillance. CAC lesions arise proximally, younger (median 45–50 years), and synchronously, complicating detection (18, 21).

### 3.2 Genetic and epigenetic alterations

Genetic and epigenetic instability is a central feature of CAC. Unlike sporadic CRC, where APC mutations are early initiating events, CAC is characterized by early and frequent alterations in TP53, often detectable in non-dysplastic mucosa (24). TP53 mutations confer resistance to apoptosis and promote clonal survival under inflammatory stress. In contrast, APC alterations typically occur later in CAC progression highlighting fundamental differences in tumor initiation (25).

Chronic inflammation accelerates the accumulation of somatic mutations through increased epithelial turnover and impaired DNA repair mechanisms (26). Copy number alterations, chromosomal instability and aneuploidy are common and may precede histologic dysplasia (26). Epigenetic changes, including DNA methylation abnormalities and histone modifications further contribute to gene silencing and oncogenic activation (27). Hypermethylation of tumor suppressor genes and dysregulation of non-coding RNAs such as microRNAs, modulate pathways involved in cell cycle control, immune signaling and barrier integrity (28). Together, these genetic and epigenetic alterations create a permissive landscape for malignant transformation and offer potential avenues for biomarker development (29).

### 3.3 Inflammatory and immune pathways

Persistent immune activation is a key driver of carcinogenesis in UC. Proinflammatory cytokines such as tumor necrosis factor (TNF), interleukin-6 (IL-6), IL-1β and IL-23 sustain epithelial proliferation, inhibit apoptosis and promote angiogenesis (30). Activation of transcription factors including NF-κB and STAT3 links inflammation directly to oncogenic signaling by upregulating genes involved in survival, proliferation and invasion (31).

The adaptive immune response also plays a dual role. While immune surveillance can eliminate transformed cells, chronic inflammation promotes immune exhaustion and tolerance allowing dysplastic clones to escape detection (32). Regulatory T cells, myeloid-derived suppressor cells and alternatively activated macrophages (M2 macrophages) contribute to an immunosuppressive tumor microenvironment (32). Moreover, long-standing inflammation disrupts antigen presentation and cytotoxic T-cell function further facilitating tumor progression (33). These insights provide a mechanistic rationale for the observed association between cumulative inflammatory burden and CRC risk and help explain the protective effect of sustained mucosal healing (34).

### 3.4 Oxidative stress, barrier dysfunction and microenvironment

Oxidative and nitrosative stress represents a critical link between inflammation and DNA damage in CAC. Activated neutrophils and macrophages generate reactive oxygen and nitrogen species that induce DNA adducts, base modifications and strand breaks (35). In the setting of defective repair, these insults lead to mutational accumulation and chromosomal instability. Oxidative stress also alters signaling pathways governing cell proliferation and apoptosis, reinforcing carcinogenic processes (36).

Concomitantly, chronic inflammation disrupts epithelial barrier integrity. Loss of tight junction proteins and mucus layer depletion increase intestinal permeability, amplifying exposure to luminal antigens and microbial products (37). This perpetuates inflammation and fosters microbiota-driven genotoxicity. The tumor microenvironment in CAC is further shaped by stromal fibroblasts, endothelial cells and extracellular matrix remodeling which collectively support angiogenesis, invasion, and metastatic potential (38).

CAC emerges from the convergence of chronic inflammation, genetic and epigenetic instability, immune dysregulation, oxidative stress and microenvironmental remodeling (39). Understanding these interrelated mechanisms is essential for refining risk stratification, improving surveillance and identifying novel targets for chemoprevention and precision medicine in ulcerative colitis (5, 39).

## 4 Role of the intestinal microbiota

**Figure 2:**
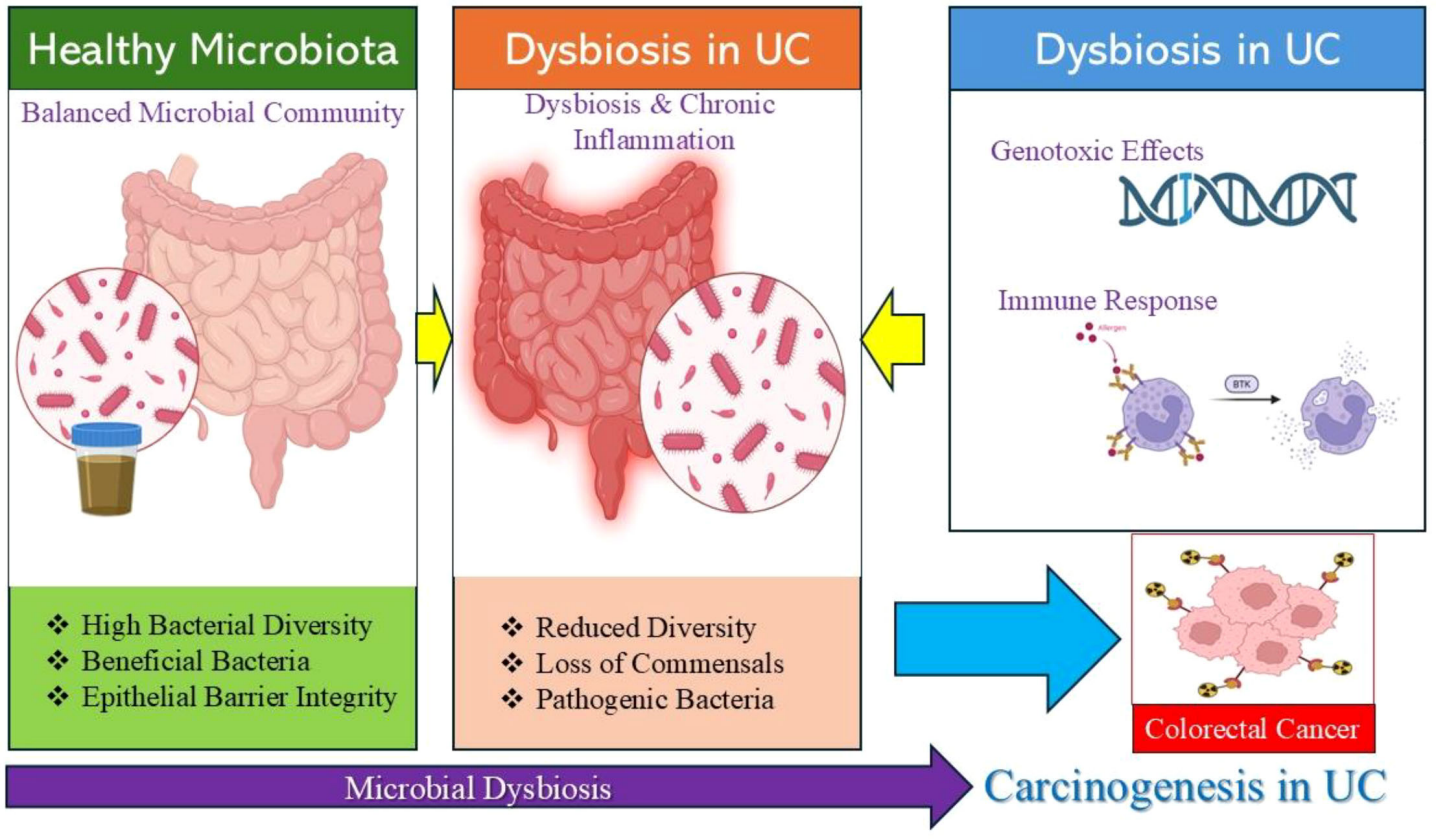
Role of microbial dysbiosis in the progression from ulcerative colitis to colorectal cancer. This schematic illustration summarizes the proposed mechanisms linking gut microbiota alterations to carcinogenesis in UC. In healthy individuals, the intestinal microbiota is characterized by high bacterial diversity, predominance of beneficial commensal bacteria and preservation of epithelial barrier integrity. In UC, microbial dysbiosis is marked by reduced microbial diversity, depletion of protective commensals and expansion of pathogenic bacteria which contribute to chronic mucosal inflammation. Dysbiosis may promote carcinogenesis through multiple mechanisms including genotoxic effects, activation of pro-inflammatory immune responses, oxidative stress and disruption of epithelial barrier function, ultimately facilitating the development of CAC.

The intestinal microbiota plays a central role in maintaining colonic homeostasis and has emerged as a critical modulator of inflammation and carcinogenesis in UC (40). The role of microbiota in UC is represented in **Figure 2**.

The healthy colon harbors a diverse and metabolically active microbial ecosystem that contributes to epithelial barrier integrity, immune tolerance and nutrient metabolism (40). In UC, this balanced ecosystem is disrupted, leading to dysbiosis characterized by reduced microbial diversity, loss of beneficial commensals and expansion of potentially pathogenic species (41). Growing evidence indicates that microbiota alterations not only perpetuate chronic inflammation but also directly contribute to the development of CAC through genotoxic, immunologic and metabolic mechanisms (40, 41).

### 4.1 Dysbiosis in UC and CAC

Patients with UC consistently exhibit a reduction in overall microbial diversity with a depletion of Firmicutes particularly short-chain fatty acid (SCFA) producing taxa such as *Faecalibacterium prausnitzii* and *Roseburia* species and an enrichment of Proteobacteria and Actinobacteria (42). This dysbiotic signature is more pronounced in patients with long standing disease and those who develop dysplasia or CRC (42). Importantly, dysbiosis in UC is not merely a consequence of inflammation but also an active driver of disease chronicity and carcinogenesis (43).

In CAC, microbial communities show distinct compositional and functional changes compared with both healthy controls and sporadic CRC. Studies have demonstrated enrichment of bacteria with pro-inflammatory or genotoxic properties, suggesting a microbiota-driven “oncogenic niche” (44). These alterations may occur early, preceding histologic dysplasia and can extend across large areas of inflamed mucosa, reinforcing the concept of field cancerization (44).

### 4.2 Microbial induced genotoxicity

One of the most compelling links between the microbiota and CAC is the capacity of certain bacteria to directly induce DNA damage (45). Several microbial species harbor virulence factors or metabolic pathways that generate genotoxins which can promote mutagenesis in colonic epithelial cells (45). For example, *E. coli* strains carrying the polyketide synthase (pks) island produce colibactin (46). These pks^+^ *E. coli* strains are enriched in inflamed mucosa and have been implicated in both sporadic CRC and CAC (47). Recent mechanistic studies have demonstrated that colibactin produced by pks^+^ *E. coli* induces DNA interstrand crosslinks and double-strand breaks, generating distinct mutational signatures that have been identified in a subset of colorectal cancers, thereby providing direct molecular evidence linking bacterial genotoxicity to tumorigenesis (48, 49). Recent work also highlights regulatory pathways influencing colibactin activity and potential strategies to mitigate its genotoxic effects in the intestinal environment (50, 51).

Other microbes contribute indirectly to genotoxic stress by generating reactive oxygen and nitrogen species or by stimulating host immune cells to do so (52). In the inflamed UC colon, activated neutrophils and macrophages release reactive intermediates in response to microbial stimuli, amplifying oxidative DNA damage (52). Repeated exposure to these insults, coupled with impaired DNA repair in chronically inflamed epithelium, accelerates the accumulation of oncogenic mutations (52).

### 4.3 Microbiota-immune interactions and chronic inflammation

The intestinal microbiota exerts profound effects on mucosal immunity shaping both innate and adaptive immune responses (53). In UC, dysbiosis disrupts immune tolerance and promotes sustained inflammatory signaling a key driver of CAC (54). Pattern recognition receptors, such as Toll-like receptors (TLRs) and nucleotide-binding oligomerization domain (NOD)-like receptors detect microbial-associated molecular patterns and activate downstream pathways including NF-κB and inflammasomes (55). Persistent activation of these pathways leads to chronic cytokine production, epithelial proliferation and resistance to apoptosis (55).

Specific microbial taxa have been shown to skew immune responses toward pro-tumorigenic phenotypes (56). For instance, enrichment of *Fusobacterium nucleatum* has been associated with increased expression of inflammatory cytokines, recruitment of myeloid-derived suppressor cells and suppression of cytotoxic T-cell activity (56). Although *F. nucleatum* is best studied in sporadic CRC, emerging evidence suggests a similar role in CAC, particularly in advanced lesions (57). Conversely, loss of anti-inflammatory commensals reduces regulatory T-cell induction and SCFA-mediated immune regulation, further tipping the balance toward carcinogenic inflammation (58).

### 4.4 Microbial metabolites and carcinogenesis

Beyond microbial composition, functional alterations in microbial metabolism are increasingly recognized as key determinants of CAC risk (59). SCFAs, particularly butyrate, play a protective role by serving as an energy source for colonocytes, reinforcing epithelial barrier function and exerting anti-inflammatory and anti-proliferative effects (59). Butyrate also acts as a histone deacetylase inhibitor, influencing gene expression and promoting apoptosis of transformed cells (60). In UC, depletion of butyrate-producing bacteria leads to reduced SCFA availability and weakening these protective mechanisms (59, 60).

In contrast, dysbiotic microbiota may produce metabolites that promote carcinogenesis. Secondary bile acids such as deoxycholic acid, can induce oxidative stress, DNA damage and pro-inflammatory signaling (61). Patients with UC, particularly those with concomitant PSC exhibit altered bile acid pools that interact with the microbiota to enhance epithelial injury and tumorigenesis (62). Additional microbial metabolites, including hydrogen sulfide and polyamines can impair mitochondrial function, disrupt DNA integrity and stimulate epithelial proliferation (63).

### 4.5 Barrier dysfunction and microbial translocation

Intestinal barrier dysfunction is both a cause and consequence of microbiota alterations in UC. Chronic inflammation leads to disruption of tight junctions, mucus layer thinning and goblet cell depletion allowing increased microbial contact with the epithelium (64). This enhanced exposure facilitates bacterial translocation and amplifies immune activation creating a self-reinforcing cycle of inflammation and dysbiosis (65).

Barrier defects also enable microbial products such as lipopolysaccharide and flagellin, to penetrate the lamina propria and activate oncogenic signaling pathways (66). In experimental models, increased microbial translocation accelerates dysplasia and carcinoma development, whereas barrier restoration mitigates tumor risk (66). These findings underscore the importance of epithelial integrity in modulating microbiota-driven carcinogenesis (67).

### 4.6 Interactions with host genetics and epigenetics

The impact of the microbiota on CAC is modulated by host genetic and epigenetic factors (68). Variants in genes involved in microbial sensing, autophagy and barrier function influence susceptibility to dysbiosis and inflammation-driven cancer (68). Conversely, microbial metabolites can shape the host epigenome through DNA methylation and histone modification, thereby altering gene expression programs relevant to carcinogenesis (70). This bidirectional interaction highlights the microbiota as an interface between environmental exposures and host biology in UC associated CRC (69).

### 4.7 Therapeutic and preventive implications

Recognition of the microbiota’s role in CAC has important clinical implications (71). Effective control of mucosal inflammation through medical therapy indirectly restores microbial balance and reduces cancer risk (71). Emerging strategies aimed at directly modulating the microbiota including dietary interventions, probiotics, prebiotics, postbiotics and fecal microbiota transplantation are being actively investigated, although robust evidence for CRC prevention in UC remains limited (72).

Certain medications used in UC may exert chemopreventive effects partly through microbiota modulation. For example, 5-aminosalicylates and biologics can reduce pro-inflammatory microbial signatures by inducing mucosal healing (73). Ursodeoxycholic acid, particularly in UC with PSC alters bile acid microbiota interactions and has been associated with reduced dysplasia risk in some studies (73, 74).

## 5 Molecular and histologic biomarkers

Accurate risk stratification and early detection of CAC remain major challenges in UC given the often flat, multifocal and inflammation-masked nature of dysplasia (75). While surveillance colonoscopy is the cornerstone of prevention, there is growing recognition that histologic and molecular biomarkers can complement endoscopic assessment by identifying patients at heightened cancer risk, predicting progression and guiding personalized surveillance and chemoprevention strategies (76).

### 5.1 Histologic biomarkers

The presence and grade of dysplasia are the strongest predictors of CRC risk. LGD is associated with a substantially increased risk of progression to HGD or CRC, particularly when lesions are non-polypoid, multifocal, or invisible endoscopically (77). HGD carries a high likelihood of synchronous or imminent carcinoma and often warrants colectomy (77).

Recent international consensus statements and updated guideline discussions have also emphasized the importance of recognizing nonconventional dysplasia in inflammatory bowel disease-associated neoplasia (78). These morphologic subtypes including hypermucinous, crypt cell, goblet cell deficient and serrated-like dysplasia may be underrecognized but are increasingly considered clinically relevant due to their potential association with neoplastic progression and diagnostic challenges in routine pathology practice (78, 79). Updated guidance aligned with recent SCENIC recommendations highlights the need for careful histologic evaluation and multidisciplinary correlation when these patterns are identified during surveillance (78, 79).

Beyond overt dysplasia, chronic inflammatory burden itself is a powerful histologic biomarker. Multiple cohort studies have demonstrated that cumulative histologic inflammation assessed across serial biopsies over time is independently associated with CRC risk, even after adjustment for disease duration and extent (80). Persistent neutrophilic activity, basal plasmacytosis, crypt architectural distortion and ulceration reflect ongoing epithelial injury and regenerative stress that promote carcinogenesis (80). Consequently, histologic remission is increasingly recognized as a meaningful therapeutic target with potential chemopreventive implications (77, 80).

Additional histologic features, such as mucin depletion, Paneth cell metaplasia in the distal colon and epithelial aneuploidy detected by flow cytometry or image analysis, have been linked to neoplastic risk (81). Aneuploidy can be detected in non-dysplastic mucosa and may precede visible dysplasia, making it an attractive early warning marker, although its use remains largely confined to research settings (82).

### 5.2 Molecular biomarkers

Molecular alterations precede histologic dysplasia in CAC offering opportunities for earlier risk prediction (83). Among genetic biomarkers, TP53 mutations are the most consistently identified and clinically relevant (84). Unlike sporadic CRC, where TP53 alterations occur late, TP53 mutations and protein overexpression can be detected in non-dysplastic or inflamed mucosa in UC and are strongly associated with progression to dysplasia and carcinoma. Immunohistochemical staining for p53 is widely available and increasingly used as an adjunct in dysplasia assessment (84).

Chromosomal instability and copy number alterations represent additional molecular hallmarks of CAC. Detection of DNA aneuploidy, loss of heterozygosity and broad copy number changes correlates with future neoplasia risk (85). Advances in next-generation sequencing (NGS) have enabled more comprehensive mutational profiling, revealing early involvement of genes related to cell cycle control, DNA repair, and immune signaling (85).

Epigenetic biomarkers are also gaining prominence. Aberrant DNA methylation patterns, including hypermethylation of tumor suppressor genes and global hypomethylation, accumulate with disease duration and inflammatory burden (86). Panels of methylated genes detectable in tissue or stool samples have shown promise for identifying high-risk patients, although standardization and validation are ongoing (87). Dysregulated microRNAs further contribute to carcinogenesis by modulating inflammatory and oncogenic pathways and may serve as minimally invasive biomarkers (87).

### 5.3 Emerging multimodal and non-invasive biomarkers

Recent efforts have focused on integrating molecular biomarkers with histology, endoscopy and clinical features to improve predictive accuracy (88). Stool-based biomarkers, including methylated DNA, microbial signatures and inflammatory mediators offer a non-invasive approach to risk assessment and surveillance augmentation (89). Similarly, transcriptomic and proteomic profiling of mucosal biopsies may identify high-risk inflammatory states even in the absence of dysplasia (88, 89).

Despite their promise, most molecular biomarkers are not yet ready for routine clinical use due to cost, technical complexity and limited prospective validation. However, rapid advances in sequencing technologies and bioinformatics are likely to accelerate their translation into practice (90).

### 5.4 Clinical implications

The incorporation of histologic and molecular biomarkers into clinical decision-making has the potential to transform CRC prevention in UC (91). Biomarker-informed risk stratification could identify patients who benefit from intensified surveillance or early surgery, while sparing low-risk individuals from unnecessary procedures (91). Ultimately, combining biomarkers with precision endoscopy and effective inflammation control may enable a more personalized and proactive approach to CAC prevention (91). The key histologic and molecular biomarkers in CAC are represented in **Table 1**.

**Table 1:** Key histologic and molecular biomarkers in CAC cancer.

Biomarker category	Specific marker	Clinical significance	Current clinical use	References
Histologic	LGD	Moderate-high risk of progression to HGD or CRC; risk higher when lesions are non-polypoid, multifocal, or endoscopically invisible	Routine pathology	(92)
Histologic	HGD	Very high risk of synchronous or imminent CRC; often indicates need for colectomy or advanced endoscopic management	Routine pathology	(93)
Histologic	Cumulative inflammatory burden	Independent predictor of CRC risk; persistent moderate-severe histologic inflammation across surveillance biopsies associated with increased long-term neoplasia risk	Increasingly recognized	(94)
Histologic	Aneuploidy	Early marker of genomic instability; may precede visible dysplasia and indicate increased neoplastic potential	Research/limited use	(95)
Genetic	TP53 mutation/p53 overexpression	Early molecular alteration in CAC; associated with dysplasia progression and field cancerization	Adjunctive pathology	(96)
Genetic	Copy number alterations	Reflect chromosomal instability and clonal expansion associated with dysplasia progression	–	(97)
Epigenetic	DNA methylation panels	Potential early detection and risk stratification biomarker; may identify high-risk mucosa before morphologic dysplasia	Emerging	(98)
Epigenetic	MicroRNA dysregulation	Regulates inflammatory and oncogenic signaling pathways linked to CAC development	Research	(99)
Non-invasive	Stool DNA/microbial markers	Potential non-invasive adjunct for detecting neoplastic or dysplastic changes during surveillance	Investigational	(100)

## 6 Surveillance strategies in ulcerative colitis

Surveillance for CRC is a cornerstone of long-term management in UC aiming to detect dysplasia at an early, curable stage and thereby reduce CRC-related morbidity and mortality (22). Owing to the unique biology of CAC characterized by flat, multifocal and inflammation associated lesions surveillance strategies in UC differ substantially from those applied to the general population (22). Advances in endoscopic technology and a growing emphasis on individualized risk stratification have reshaped contemporary surveillance paradigms (22).

### 6.1 Contemporary surveillance techniques

High quality colonoscopy is the foundation of CRC surveillance in UC. Key procedural elements include excellent bowel preparation, careful inspection during withdrawal and biopsies obtained under conditions of quiescent disease whenever possible (101). High-definition white-light endoscopy (HD-WLE) has largely replaced standard-definition colonoscopy and significantly improves mucosal visualization and dysplasia detection (101).

Dye-based chromoendoscopy (CE) using topical application of indigo carmine or methylene blue is now considered the preferred surveillance technique by many international guidelines (102). CE enhances visualization of subtle mucosal abnormalities and improves detection of flat and non-polypoid dysplasia, which are common in UC (103). Randomized trials and meta-analyses have demonstrated superior dysplasia detection rates with CE compared with conventional white-light endoscopy particularly in experienced hands (103). As a result, targeted biopsies of visually abnormal areas during CE have largely supplanted the historical practice of extensive random biopsies (102, 103).

Virtual chromoendoscopy techniques including narrow-band imaging (NBI), i-scan, and blue light imaging offer an alternative to dye-based methods (104). These technologies enhance mucosal and vascular patterns without the need for dye application reducing procedure time and increasing practicality (105). While early studies suggested inferior performance compared with dye-based CE, more recent data using high-definition platforms indicate comparable dysplasia detection rates, leading some guidelines to endorse virtual chromoendoscopy as an acceptable alternative when dye-based CE is unavailable (105).

Targeted biopsies of visible lesions are now favored over random sampling in most surveillance settings (104, 106). Random biopsies may still have a role in selected high-risk populations such as patients with PSC or those with a history of invisible dysplasia, where lesions may be particularly subtle or multifocal (106). Endoscopic resection of well-demarcated dysplastic lesions is increasingly feasible, provided complete excision can be achieved and surrounding mucosa is carefully assessed (106).

### 6.2 Risk-adapted surveillance intervals and practical challenges

Contemporary surveillance guidelines advocate a risk-adapted approach to colonoscopy intervals reflecting the heterogeneity of CRC risk among UC patients (107). Surveillance typically begins 8–10 years after disease onset in patients with colitis extending beyond the rectum or immediately at diagnosis in those with concomitant PSC (107). Interval recommendations range from annual to five-yearly colonoscopy, depending on individual risk factors (107).

High risk patients such as those with PSC, prior dysplasia, extensive colitis with moderate to severe ongoing inflammation or a strong family history of CRC generally require annual surveillance. Intermediate-risk individuals including those with extensive colitis in sustained remission or left-sided colitis with additional risk modifiers are often advised surveillance every 2–3 years (108). Low-risk patients with limited disease extent and long-standing histologic remission may safely undergo less frequent surveillance at 3-5-year intervals (108).

Despite clear guideline frameworks that real world implementation of risk adapted surveillance faces several challenges. Accurate assessment of cumulative inflammatory burden requires longitudinal histologic data that may not always be readily available (108, 109). Interobserver variability in dysplasia interpretation, particularly for low grade or indefinite lesions which can complicate clinical decision making and underscores the importance of expert gastrointestinal pathology review. Access to chromoendoscopy expertise and advanced imaging technologies varies widely across healthcare settings which potentially limiting equitable care (109).

Patient related factors including adherence to surveillance recommendations, procedural tolerance and competing comorbidities which further influence surveillance effectiveness (111). In older patients or those with significant comorbidity, the balance between surveillance benefit and procedural risk must be carefully considered. Additionally, uncertainty persists regarding optimal management after endoscopic resection of dysplasia and the duration of intensified follow-up (111).

Overall, surveillance strategies in UC are evolving toward a more personalized model that integrates endoscopic advances with individualized risk assessment (14). Continued efforts to standardize practice, improve access to high quality surveillance and incorporate emerging biomarkers are essential to further reduce CRC risk in this population (5, 14).

## 7 Chemoprevention: current agents and emerging strategies

Chemoprevention represents an important adjunct to surveillance colonoscopy in reducing the risk of CAC in UC (110). While sustained control of inflammation remains the central preventive strategy and accumulating evidence suggests that several pharmacologic and non-pharmacologic agents may modify CRC risk through anti-inflammatory, immunomodulatory and molecular mechanisms (110). However, much of the evidence derives from observational studies with heterogeneous designs, variable follow-up durations, and potential confounding factors such as disease severity, treatment adherence, and surveillance intensity. These limitations should be considered when interpreting reported chemopreventive effects.

### 7.1 Conventional UC therapies with chemopreventive effects

Conventional UC therapies may exert chemopreventive effects primarily by achieving durable mucosal healing and reducing cumulative inflammatory burden (110). Among these, 5-aminosalicylates (5-ASA) are the most extensively studied. Multiple observational studies and meta-analyses suggest that long-term 5-ASA use is associated with a reduced risk of CRC in UC, particularly at higher cumulative doses and with good adherence (73). Proposed mechanisms include inhibition of cyclooxygenase-2, scavenging of reactive oxygen species and modulation of nuclear factor-κB signaling (73). However, the magnitude of this protective effect remains debated. Some cohort studies and systematic reviews have reported inconsistent results, and the apparent benefit may partly reflect confounding by indication, differences in surveillance practices, or improved adherence among patients with milder disease. Consequently, although 5-ASA remains widely considered to have potential chemopreventive properties, the strength of causal evidence remains uncertain (112).

Immunomodulators such as thiopurines may indirectly reduce CAC risk by maintaining steroid-free remission and limiting chronic inflammation (113). Some population-based studies demonstrate a modest reduction in CRC incidence among thiopurine exposed patients (114). Nevertheless, these findings must be interpreted cautiously because long-term thiopurine therapy is also associated with increased risks of lymphoma and non-melanoma skin cancer, which complicates their risk-benefit profile in chemoprevention. Biologic therapies, including anti-tumor necrosis factor (TNF) agents, integrin inhibitors and interleukin-12/23 antagonists have transformed UC management (114). Emerging real-world data suggest that sustained biologic-induced mucosal healing may translate into lower CRC risk, although direct evidence linking biologic therapy to reduced CAC incidence remains limited and long-term cancer-specific outcomes are still under investigation (3) (**Table 2**).

**Table 2:** Conventional UC therapies with potential chemopreventive effects.

Agent class	Examples	Proposed mechanisms	Evidence strength	References
5-ASA	Mesalamine, sulfasalazine	Anti-inflammatory, COX-2 inhibition, antioxidant	Moderate–strong	115
Thiopurines	Azathioprine, 6-MP	Inflammation control, immune modulation	Moderate	116
Anti-TNF biologics	Infliximab, adalimumab	Mucosal healing, cytokine suppression	Emerging	117
Other biologics	Vedolizumab, ustekinumab	Targeted immune modulation	Limited/emerging	118

### 7.2 Non-conventional and adjunctive chemoprevention

Beyond standard UC therapies, several non-conventional and adjunctive agents have been explored for CRC risk reduction. Ursodeoxycholic acid (UDCA) has been studied extensively in UC patients with PSC (119). By modifying bile acid composition and reducing epithelial exposure to carcinogenic secondary bile acids, UDCA has been associated with a reduced risk of dysplasia and CRC in some observational studies. Although results are inconsistent and dose-dependent effects have been reported (119).

Statins have attracted interest due to their pleiotropic anti-inflammatory, pro-apoptotic and anti-proliferative properties (120). Several population-based studies suggest a lower CRC risk among statin users with IBD, but randomized data are lacking (120). Nonsteroidal anti-inflammatory drugs (NSAIDs) and aspirin are effective chemopreventive agents in sporadic CRC (119, 120). However, their role in UC is limited by gastrointestinal toxicity and the risk of disease exacerbation (120).

Dietary interventions, probiotics and microbiota-directed therapies represent emerging areas of interest (121). Diets rich in fiber and nutrients containing anti-inflammatory may indirectly reduce CRC risk through microbiota modulation and SCFA production, though high-quality evidence is limited (121, 122). Experimental agents targeting oxidative stress, immune checkpoints, or epigenetic pathways are under investigation but remain far from clinical application (122) (**Table 3**).

**Table 3:** Non-conventional and adjunctive chemopreventive strategies.

Strategy	Target population	Proposed mechanism	Current status	References
UDCA	UC with PSC	Bile acid modulation	Selective use	(123)
Statins	UC ± cardiovascular risk	Anti-inflammatory, pro-apoptotic	Observational evidence	(124)
Aspirin/NSAIDs	Selected patients	COX inhibition	Limited by toxicity	(125)
Diet/microbiota	All UC patients	Barrier and immune modulation	Emerging	(54)

### 7.3 Balancing benefits, risks, and adherence

The implementation of chemoprevention in UC requires careful balancing of potential benefits against drug-related risks, patient preferences and long-term adherence (126). While 5-ASA agents are generally safe and well tolerated, adherence declines over time, potentially diminishing their protective effect (126). Thiopurines and biologics offer potent inflammation control but carry risks of infection and malignancy that must be weighed against their indirect chemopreventive benefits (127).

Importantly, no chemopreventive agent can substitute for effective surveillance colonoscopy. Instead, chemoprevention should be viewed as a complementary strategy within a comprehensive, risk-adapted prevention framework (128). Shared decision-making is essential, particularly when considering adjunctive agents with limited or conflicting evidence (128).

## 8 Precision medicine and future directions

The paradigm of CRC prevention in UC is progressively shifting toward precision medicine, integrating clinical, endoscopic, molecular and microbial data to individualize risk assessment and preventive strategies. Traditional surveillance models based largely on disease duration and extent are increasingly recognized as insufficient to capture the heterogeneity of CAC risk. Precision approaches aim to identify high-risk patients earlier, optimize surveillance intensity and tailor therapeutic and chemopreventive interventions (69, 129).

Advances in multi-omics technologies have accelerated progress in this field. Genomic and epigenomic profiling of colonic mucosa can detect early oncogenic alterations, such as TP53 mutations, chromosomal instability and aberrant DNA methylation, even in histologically non-dysplastic tissue. Transcriptomic and proteomic signatures reflecting cumulative inflammatory burden and immune dysregulation may further refine risk prediction. In parallel, microbiome-based profiling is emerging as a promising tool with specific microbial taxa and metabolite patterns linked to dysplasia and CRC development (11, 17, 40).

Artificial intelligence and machine learning are poised to enhance both endoscopic detection and risk stratification. AI-assisted image analysis can improve real time dysplasia recognition during colonoscopy while predictive models integrating longitudinal clinical and molecular data may support personalized surveillance intervals. Additionally, digital pathology and centralized expert review may reduce interobserver variability in dysplasia interpretation.

Future preventive strategies are likely to combine precision surveillance with targeted chemoprevention and optimized inflammation control. Biomarker-guided therapy selection, adaptive surveillance intervals, and microbiota-modulating interventions represent key areas of ongoing research. Robust prospective validation and cost-effectiveness analyses will be essential before widespread clinical adoption.

## 9 Conclusions

CRC remains a serious but increasingly preventable complication of UC. Contemporary evidence demonstrates that while the relative risk of CRC in UC persists. The absolute risk has declined markedly due to improved disease control, advanced endoscopic surveillance and evolving preventive strategies. CAC arises through a distinct inflammation-driven pathway, underscoring the importance of sustained mucosal healing and cumulative inflammatory control. Advances in chromoendoscopy, risk-adapted surveillance intervals and endoscopic management of dysplasia have refined clinical practice; while emerging molecular, histologic and microbiota-based biomarkers offer new avenues for early risk stratification. Chemoprevention, particularly through effective anti-inflammatory therapy, provides an important adjunct to surveillance. Although careful consideration of benefits, risks and adherence is essential. Looking forward, precision medicine approaches integrating clinical, endoscopic and multi-omics data hold promise for individualized surveillance and prevention. Continued translational research and prospective validation are critical to further reducing CRC morbidity and mortality in patients with UC.

## References

[1] Gajendran M Loganathan P Jimenez G Catinella AP Ng N Umapathy C . A comprehensive review and update on ulcerative colitis. Dis Mon. (2019) 65:100851. doi: 10.1016/j.disamonth.2019.02.004. PMID: 30837080

[2] Zamani M Alizadeh-Tabari S . Does elderly-onset inflammatory bowel disease increase risk of colorectal cancer? A systematic review and meta-analysis. J Clin Med. (2023) 13:148. doi: 10.3390/jcm13010148. PMID: 38202155 PMC10779516

[3] Nardone OM Zammarchi I Santacroce G Ghosh S Iacucci M . Inflammation-driven colorectal cancer associated with colitis: from pathogenesis to changing therapy. Cancers. (2023) 15:2389. doi: 10.3390/cancers15082389. PMID: 37190315 PMC10136846

[4] Klepp P Brackmann S Cvancarova M Hoivik ML Hovde Ø Henriksen M . Risk of colorectal cancer in a population-based study 20 years after diagnosis of ulcerative colitis: Results from the IBSEN study. BMJ Open Gastroenterol. (2020) 7:e000361. doi: 10.1136/bmjgast-2019-000361. PMID: 32337058 PMC7170403

[5] Li W Zhao T Wu D Li J Wang M Sun Y . Colorectal cancer in ulcerative colitis: mechanisms, surveillance and chemoprevention. Curr Oncol. (2022) 29:6091–114. doi: 10.3390/curroncol29090479. PMID: 36135048 PMC9498229

[6] Rachma B Pamungkas RP Sandra DY Sutanto H Fetarayani D . The immunological nexus between inflammatory bowel disease and colorectal cancer: a molecular perspective. Clin Immunol Commun. (2025) 8:138–58. doi: 10.1016/j.clicom.2025.11.004. PMID: 41878731

[7] Wang Y Wang P Shao L . Correlation of ulcerative colitis and colorectal cancer: a systematic review and meta-analysis. J Gastrointest Oncol. (2021) 12:2814–22. doi: 10.21037/jgo-21-624. PMID: 35070409 PMC8748039

[8] Van Munster KN Bergquist A Ponsioen CY . Inflammatory bowel disease and primary sclerosing cholangitis: One disease or two? J Hepatol. (2023) 80:155–68. doi: 10.1016/j.jhep.2023.09.031. PMID: 37940453

[9] Krzyszczyk P Acevedo A Davidoff EJ Timmins LM Marrero-Berrios I Patel M . The growing role of precision and personalized medicine for cancer treatment. TECHNOLOGY. (2018) 06:79–100. doi: 10.1142/s2339547818300020. PMID: 30713991 PMC6352312

[10] Porter RJ Arends MJ Churchhouse AMD Din S . Inflammatory bowel disease-associated colorectal cancer: translational risks from mechanisms to medicines. J Crohns Colitis. (2021) 15:2131–41. doi: 10.1093/ecco-jcc/jjab102. PMID: 34111282 PMC8684457

[11] Liu Y Ohashi Y Ushijima T . How chronic inflammation fuels carcinogenesis as an environmental epimutagen. Discover Oncol. (2025) 16:1150. doi: 10.1007/s12672-025-02971-9. PMID: 40533660 PMC12176710

[12] Ikebata A Shimoda M Okabayashi K Uraoka T Maehata T Sugimoto S . Demarcated redness associated with increased vascular density/size: A useful marker of flat-type dysplasia in patients with ulcerative colitis. Endosc Int Open. (2021) 09:E552–61. doi: 10.1055/a-1352-2709. PMID: 33860072 PMC8041573

[13] Garg P Malhotra J Kulkarni P Horne D Salgia R Singhal SS . Emerging therapeutic strategies to overcome drug resistance in cancer cells. Cancers. (2024) 16:2478. doi: 10.3390/cancers16132478. PMID: 39001539 PMC11240358

[14] Roseira J Estevinho MM Gros B Marafini I Solitano V Sousa P . Advances in endoscopy in IBD diagnostics and management. Best Pract Res Clin Gastroenterol. (2025) 78:102055. doi: 10.1016/j.bpg.2025.102055. PMID: 41350091

[15] Kefayat A Porter RJ Churchhouse AM Blackwell J Watson EF Morris AJ . Reduced risk of colorectal cancer with non-sulfasalazine 5-ASAs in ulcerative colitis and Crohn’s disease and anti-TNF therapy in ulcerative colitis: A systematic review and meta-analysis. Frontline Gastroenterol. (2025) 2025:flgastro-2025. doi: 10.1136/flgastro-2025-103409. PMID: 41802225

[16] Xie M Zheng J Yu Y Yang Q Zhou Z Xue J . Gut symbiont-derived ursodeoxycholic acid promotes fatty acid oxidation to protect against renal ischemia-reperfusion injury. Cell Rep Med. (2025) 6:102373. doi: 10.1016/j.xcrm.2025.102373. PMID: 41015035 PMC12629832

[17] Zhang L Zhang X Su T Xiao T Xu H Zhao S . Colorectal cancer risk in ulcerative colitis: an updated population-based systematic review and meta-analysis. EClinicalMedicine. (2025) 84:103269. doi: 10.1016/j.eclinm.2025.103269. PMID: 40496880 PMC12150054

[18] Catalano M Mini E Nobili S Vascotto IA Ravizza D Amorosi A . Ulcerative colitis and colorectal cancer: Pathogenic insights and precision strategies for prevention and treatment. World J Gastrointest Oncol. (2025) 17:108514. doi: 10.4251/wjgo.v17.i10.108514. PMID: 41114102 PMC12531786

[19] Yashiro M . Ulcerative colitis-associated colorectal cancer. World J Gastroenterol. (2014) 20:16389. doi: 10.3748/wjg.v20.i44.16389. PMID: 25469007 PMC4248182

[20] Everhov ÅH Askling J Söderling J Halfvarson J Eriksson J Smedby KE . Cancer incidence in patients with ulcerative colitis naïve to or treated with thiopurine and targeted therapies-a cohort study 2007 to 2022 with comparison to the general population. J Crohns Colitis. (2025) 19. doi: 10.1093/ecco-jcc/jjaf091. PMID: 40455688 PMC12203221

[21] Dregelies T Haumaier F Sterlacci W Backert S Vieth M . Mutational analysis differentiating sporadic carcinomas from colitis-associated colorectal carcinomas. Cell Commun Signal. (2024) 22:483. doi: 10.1186/s12964-024-01856-8. PMID: 39390564 PMC11465924

[22] Parra-Izquierdo V Otero-Regino W Juliao-Baños F Frías-Ordoñez JS Ibañez-Pinilla E Gil-Parada FL . Dysplasia and colorectal cancer surveillance in ulcerative colitis patients in Latin America: real-world data. J Crohns Colitis. (2024) 360:1–11. doi: 10.1093/crocol/otae081. PMID: 39834355 PMC11744193

[23] Kapadia A Joshi D Chavda A Bhatt P . From inflammation to carcinogenesis: Distinct pathways and clinical implications of IBD-associated colorectal cancer compared with sporadic CRC. Pathol Res Pract. (2025) 275:156249. doi: 10.1016/j.prp.2025.156249. PMID: 41043201

[24] Carethers JM Jung BH . Genetics and genetic biomarkers in sporadic colorectal cancer. Gastroenterology. (2015) 149:1177–1190.e3. doi: 10.1053/j.gastro.2015.06.047. PMID: 26216840 PMC4589489

[25] Tornesello M . TP53 mutations in cancer: Molecular features and therapeutic opportunities (Review). Int J Mol Med. (2024) 55. doi: 10.3892/ijmm.2024.5448. PMID: 39450536 PMC11554381

[26] Lin R Zhang C Zheng J Tian D Lei Z Chen D . Chronic inflammation-associated genomic instability paves the way for human esophageal carcinogenesis. Oncotarget. (2016) 7:24564–71. doi: 10.18632/oncotarget.8356. PMID: 27028857 PMC5029723

[27] Sadida HQ Abdulla A Marzooqi SA Hashem S Macha MA Akil ASA . Epigenetic modifications: key players in cancer heterogeneity and drug resistance. Transl Oncol. (2023) 39:101821. doi: 10.1016/j.tranon.2023.101821. PMID: 37931371 PMC10654239

[28] Parikh D Shah M . Targeting non-coding RNAs to overcome resistance and improving outcomes in glioblastoma. Glob Med Genet. (2025) 12:100075. doi: 10.1016/j.gmg.2025.100075. PMID: 41451392 PMC12731266

[29] Manoukian P Kuhnen LC Van Laarhoven HW Bijlsma MF . Association of epigenetic landscapes with heterogeneity and plasticity in pancreatic cancer. Crit Rev Oncol Hematol. (2024) 206:104573. doi: 10.1016/j.critrevonc.2024.104573. PMID: 39581245

[30] Nakase H Shimomori Y . Association of chronic inflammation-associated cancer with cytokines. Cureus. (2025) 17:e92239. doi: 10.7759/cureus.92239. PMID: 41089104 PMC12517737

[31] Zhang T Ma C Zhang Z Zhang H Hu H . NF‐κB signaling in inflammation and cancer. MedComm. (2021) 2:618–53. doi: 10.1002/mco2.104. PMID: 34977871 PMC8706767

[32] Peña-Romero AC Orenes-Piñero E . Dual effect of immune cells within tumour microenvironment: pro- and anti-tumour effects and their triggers. Cancers. (2022) 14:1681. doi: 10.3390/cancers14071681. PMID: 35406451 PMC8996887

[33] Zhao H Wu L Yan G Chen Y Zhou M Wu Y . Inflammation and tumor progression: signaling pathways and targeted intervention. Signal Transduct Target Ther. (2021) 6:263. doi: 10.1038/s41392-021-00658-5. PMID: 34248142 PMC8273155

[34] Choi CR Bakir IA Ding N Lee G Askari A Warusavitarne J . Cumulative burden of inflammation predicts colorectal neoplasia risk in ulcerative colitis: A large single-centre study. Gut. (2017) 68:414–22. doi: 10.1136/gutjnl-2017-314190. PMID: 29150489 PMC6581019

[35] Nilsson R Liu N . Nuclear DNA damages generated by reactive oxygen molecules (ROS) under oxidative stress and their relevance to human cancers, including ionizing radiation-induced neoplasia part I: physical, chemical and molecular biology aspects. Radiat Med Prot. (2020) 1:140–52. doi: 10.1016/j.radmp.2020.09.002. PMID: 41878731

[36] Zhang S Xiao X Yi Y Wang X Zhu L Shen Y . Tumor initiation and early tumorigenesis: molecular mechanisms and interventional targets. Signal Transduct Target Ther. (2024) 9:149. doi: 10.1038/s41392-024-01848-7. PMID: 38890350 PMC11189549

[37] Di Vincenzo F Del Gaudio A Petito V Lopetuso LR Scaldaferri F . Gut microbiota, intestinal permeability, and systemic inflammation: A narrative review. Intern Emerg Med. (2023) 19:275–93. doi: 10.1007/s11739-023-03374-w. PMID: 37505311 PMC10954893

[38] Han J Kim E Kim BY Soung N . Cancer-associated fibroblasts arising from endothelial-to-mesenchymal transition: Induction factors, functional roles, and transcriptomic evidence. Biology. (2025) 14:1403. doi: 10.3390/biology14101403. PMID: 41154806 PMC12561803

[39] Gharib E Robichaud GA . From crypts to cancer: A holistic perspective on colorectal carcinogenesis and therapeutic strategies. Int J Mol Sci. (2024) 25:9463. doi: 10.3390/ijms25179463. PMID: 39273409 PMC11395697

[40] Zhang H Lee BJY Wang T Xiang X Tan Y Han Y . Microbiota, chronic inflammation, and health: The promise of inflammatome and inflammatomics for precision medicine and health care. hLife. (2025) 3:307–26. doi: 10.1016/j.hlife.2025.04.004. PMID: 41878731

[41] Shen Y Fan N Ma S Cheng X Yang X Wang G . Gut microbiota dysbiosis: pathogenesis, diseases, prevention, and therapy. MedComm. (2025) 6:e70168. doi: 10.1002/mco2.70168. PMID: 40255918 PMC12006732

[42] Chulenbayeva L Jarmukhanov Z Kaliyekova K Kozhakhmetov S Kushugulova A . Quantitative alterations in short-chain fatty acids in inflammatory bowel disease: A systematic review and meta-analysis. Biomolecules. (2025) 15:1017. doi: 10.3390/biom15071017. PMID: 40723889 PMC12292850

[43] Santana PT Rosas SLB Ribeiro BE Marinho Y De Souza HSP . Dysbiosis in inflammatory bowel disease: pathogenic role and potential therapeutic targets. Int J Mol Sci. (2022) 23:3464. doi: 10.3390/ijms23073464. PMID: 35408838 PMC8998182

[44] Grellier N Severino A ArChilei S Kim J Gasbarrini A Cammarota G . Gut microbiota in inflammation and colorectal cancer: A potential toolbox for clinicians. Best Pract Res Clin Gastroenterol. (2024) 72:101942. doi: 10.1016/j.bpg.2024.101942. PMID: 39645280

[45] Pandey H Tang DWT Wong SH Lal D . Gut microbiota in colorectal cancer: biological role and therapeutic opportunities. Cancers. (2023) 15:866. doi: 10.3390/cancers15030866. PMID: 36765824 PMC9913759

[46] Lv C Abdullah M Su C Chen W Zhou N Cheng Z . Genomic characterization of Escherichia coli with a polyketide synthase (pks) island isolated from ulcerative colitis patients. BMC Genomics. (2025) 26:19. doi: 10.1186/s12864-024-11198-x. PMID: 39780077 PMC11707995

[47] Sadeghi M Mestivier D Sobhani I . Contribution of pks+ Escherichia coli (E. coli) to colon carcinogenesis. Microorganisms. (2024) 12:1111. doi: 10.3390/microorganisms12061111. PMID: 38930493 PMC11205849

[48] Zhang G Sun D . The synthesis of the novel Escherichia coli toxin—colibactin and its mechanisms of tumorigenesis of colorectal cancer. Front Microbiol. (2024) 15:1501973. doi: 10.3389/fmicb.2024.1501973. PMID: 39744397 PMC11688353

[49] Huber AR Pleguezuelos-Manzano C Puschhof J Ubels J Boot C Saftien A . Improved detection of colibactin-induced mutations by genotoxic E. coli in organoids and colorectal cancer. Cancer Cell. (2024) 42:487–496.e6. doi: 10.1016/j.ccell.2024.02.009. PMID: 38471458

[50] Bayne C Boutard M Zaplana T Tolonen AC . L-tryptophan and copper interactions linked to reduced colibactin genotoxicity in pks+ Escherichia coli. mSystems. (2024) 9:e0099224. doi: 10.1128/msystems.00992-24. PMID: 39264195 PMC11495049

[51] Yang S Wang Z Fang C Yang M Khawaled S Bonanno S . Surface expression of antitoxin on engineered bacteria neutralizes genotoxic colibactin in the gut. Nat Microbiol. (2025) 11:53–66. doi: 10.1038/s41564-025-02177-3. PMID: 41361522 PMC12833714

[52] Burgos-Molina AM Santana TT Redondo M Romero MJB . The crucial role of inflammation and the immune system in colorectal cancer carcinogenesis: A comprehensive perspective. Int J Mol Sci. (2024) 25:6188. doi: 10.3390/ijms25116188. PMID: 38892375 PMC11172443

[53] Zheng D Liwinski T Elinav E . Interaction between microbiota and immunity in health and disease. Cell Res. (2020) 30:492–506. doi: 10.1038/s41422-020-0332-7. PMID: 32433595 PMC7264227

[54] Popov J Caputi V Nandeesha N Rodriguez DA Pai N . Microbiota-immune interactions in ulcerative colitis and colitis associated cancer and emerging microbiota-based therapies. Int J Mol Sci. (2021) 22:11365. doi: 10.3390/ijms222111365. PMID: 34768795 PMC8584103

[55] Wicherska-Pawłowska K Wróbel T Rybka J . Toll-like receptors (TLRs), NOD-like receptors (NLRs), and RIG-I-like receptors (RLRs) in innate immunity. TLRs, NLRs, and RLRs ligands as immunotherapeutic agents for hematopoietic diseases. Int J Mol Sci. (2021) 22:13397. doi: 10.3390/ijms222413397. PMID: 34948194 PMC8704656

[56] Kowsarkhizi AS Yousefi B Rahimi A Aliramezani A . Targeting Fusobacterium nucleatum in colorectal cancer: Therapeutic strategies and future directions. Infect Agent Cancer. (2025) 20:70. doi: 10.1186/s13027-025-00676-w. PMID: 41088139 PMC12522427

[57] Sun Y Lu J Lau EYT Zeng Y Li SWL Au TH . Fusobacterium nucleatum enhances cholesterol biosynthesis in colorectal cancer via miR-130a-3p-mediated AMPK inhibition, a process counteracted by butyrate. Cancer Lett. (2025) 627:217810. doi: 10.1016/j.canlet.2025.217810. PMID: 40414519

[58] Du Y He C An Y Huang Y Zhang H Fu W . The role of short chain fatty acids in inflammation and body health. Int J Mol Sci. (2024) 25:7379. doi: 10.3390/ijms25137379. PMID: 39000498 PMC11242198

[59] Venegas DP De La Fuente MK Landskron G González MJ Quera R Dijkstra G . Short chain fatty acids (SCFAs)-mediated gut epithelial and immune regulation and its relevance for inflammatory bowel diseases. Front Immunol. (2019) 10:277. doi: 10.3389/fimmu.2019.00277. PMID: 30915065 PMC6421268

[60] Liu H Wang J He T Becker S Zhang G Li D . Butyrate: a double-edged sword for health? Adv Nutr. (2018) 9:21–9. doi: 10.1093/advances/nmx009. PMID: 29438462 PMC6333934

[61] Collins SL Stine JG Bisanz JE Okafor CD Patterson AD . Bile acids and the gut microbiota: Metabolic interactions and impacts on disease. Nat Rev Microbiol. (2022) 21:236–47. doi: 10.1038/s41579-022-00805-x. PMID: 36253479 PMC12536349

[62] Fousekis FS Mpakogiannis K Lianos GD Antonelli E Bassotti G Katsanos KH . Gut–liver axis, microbiota, bile acids, and immune response in pathogenesis of primary sclerosing cholangitis: An overview. J Clin Med. (2025) 14:7817. doi: 10.3390/jcm14217817. PMID: 41227212 PMC12608333

[63] Slade L Deane CS Szewczyk NJ Etheridge T Whiteman M . Hydrogen sulfide supplementation as a potential treatment for primary mitochondrial diseases. Pharmacol Res. (2024) 203:107180. doi: 10.1016/j.phrs.2024.107180. PMID: 38599468

[64] Dunleavy KA Raffals LE Camilleri M . Intestinal barrier dysfunction in inflammatory bowel disease: Underpinning pathogenesis and therapeutics. Dig Dis Sci. (2023) 68:4306–20. doi: 10.1007/s10620-023-08122-w. PMID: 37773554 PMC10798146

[65] Charitos IA Scacco S Cotoia A Castellaneta F Castellana G Pasqualotto F . Intestinal microbiota dysbiosis role and bacterial translocation as a factor for septic risk. Int J Mol Sci. (2025) 26:2028. doi: 10.3390/ijms26052028. PMID: 40076650 PMC11900423

[66] Ghosh SS Wang J Yannie PJ Ghosh S . Intestinal barrier dysfunction, LPS translocation, and disease development. J Endocr Soc. (2020) 4:bvz039. doi: 10.1210/jendso/bvz039. PMID: 32099951 PMC7033038

[67] Ullah S Wu C Zou X Zhong Y Ahmad A Jan AU . Understanding microbiota-driven oncogenesis: The role of metabolites in tumorigenesis. iScience. (2025) 28:113945. doi: 10.1016/j.isci.2025.113945. PMID: 41333317 PMC12666704

[68] Turcu S Grama F Gazouli M . Gut microbiome-mediated genetic and epigenetic alterations in colorectal cancer: Population-specific insights. Biomedicines. (2025) 13:2262. doi: 10.3390/biomedicines13092262. PMID: 41007823 PMC12467240

[69] Liu X Yang B Tang D . Bidirectional regulation of the gut microbiome-immune axis in the immune microenvironment of colorectal cancer and targeted interventions. World J Gastrointest Oncol. (2025) 17:109503. doi: 10.4251/wjgo.v17.i10.109503. PMID: 41114103 PMC12531810

[70] Krautkramer KA Dhillon RS Denu JM Carey HV . Metabolic programming of the epigenome: host and gut microbial metabolite interactions with host chromatin. Transl Res. (2017) 189:30–50. doi: 10.1016/j.trsl.2017.08.005. PMID: 28919341 PMC5659875

[71] Drobner JC Lichtbroun BJ Singer EA Ghodoussipour S . Examining the role of microbiota-centered interventions in cancer therapeutics: Applications for urothelial carcinoma. Technol Cancer Res Treat. (2023) 22:15330338231164196. doi: 10.1177/15330338231164196. PMID: 36938621 PMC10028658

[72] Ciernikova S Sevcikova A Drgona L Mego M . Modulating the gut microbiota by probiotics, prebiotics, postbiotics, and fecal microbiota transplantation: An emerging trend in cancer patient care. Biochim Biophys Acta Rev Cancer. (2023) 1878:188990. doi: 10.1016/j.bbcan.2023.188990. PMID: 37742728

[73] Qiu X Ma J Wang K Zhang H . Chemopreventive effects of 5-aminosalicylic acid on inflammatory bowel disease-associated colorectal cancer and dysplasia: a systematic review with meta-analysis. Oncotarget. (2016) 8:1031–45. doi: 10.18632/oncotarget.13715. PMID: 27906680 PMC5352032

[74] Wolf JM Rybicki LA Lashner BA . The impact of ursodeoxycholic acid on cancer, dysplasia and mortality in ulcerative colitis patients with primary sclerosing cholangitis. Aliment Pharmacol Ther. (2005) 22:783–8. doi: 10.1111/j.1365-2036.2005.02650.x. PMID: 16225486

[75] Savo MT De Amicis M Cozac DA Cordoni G Corradin S Cozza E . Comparative prognostic value of coronary calcium score and perivascular fat attenuation index in coronary artery disease. J Clin Med. (2024) 13:5205. doi: 10.3390/jcm13175205. PMID: 39274418 PMC11395785

[76] Pinsky PF Schoen RE . Contribution of surveillance colonoscopy to colorectal cancer prevention. Clin Gastroenterol Hepatol. (2020) 18:2937–44. doi: 10.1016/j.cgh.2020.01.037. PMID: 32017987 PMC7549191

[77] Choi CR Ignjatovic-Wilson A Askari A Lee GH Warusavitarne J Moorghen M . Low-grade dysplasia in ulcerative colitis: Risk factors for developing high-grade dysplasia or colorectal cancer. Am J Gastroenterol. (2015) 110:1461–71. doi: 10.1038/ajg.2015.248. PMID: 26416190 PMC4697133

[78] Choi W Yozu M Miller GC Shih AR Kumarasinghe P Misdraji J . Nonconventional dysplasia in patients with inflammatory bowel disease and colorectal carcinoma: A multicenter clinicopathologic study. Mod Pathol. (2019) 33:933–43. doi: 10.1038/s41379-019-0419-1. PMID: 31822800

[79] Sejben A Balajthy Z Török ZK Almási S Lantos T . Conventional and non-conventional dysplasias associated with inflammatory bowel disease—a single-centre experience. Med Sci. (2026) 14:78. doi: 10.3390/medsci14010078. PMID: 41718125 PMC12922047

[80] Yvellez OV Rai V Sossenheimer PH Hart J Turner JR Weber C . Cumulative histologic inflammation predicts colorectal neoplasia in ulcerative colitis: a validation study. Inflammation Bowel Dis. (2020) 27:203–6. doi: 10.1093/ibd/izaa047. PMID: 32152624 PMC7813748

[81] Singh R Balasubramanian I Zhang L Gao N . Metaplastic paneth cells in extra-intestinal mucosal niche indicate a link to microbiome and inflammation. Front Physiol. (2020) 11:280. doi: 10.3389/fphys.2020.00280. PMID: 32296343 PMC7138011

[82] Pessing-Shabi A Zlotogorski-Hurvitz A Yarom N Kaplan I Trakhtenbrot L Hirshberg A . Is aneuploidy a consistent marker for Malignant transformation risk in oral lichen planus? Head Neck Pathol. (2025) 19:54. doi: 10.1007/s12105-025-01779-x. PMID: 40338413 PMC12061817

[83] Gaiani F Marchesi F Negri F Greco L Malesci A De’Angelis GL . Heterogeneity of colorectal cancer progression: Molecular gas and brakes. Int J Mol Sci. (2021) 22:5246. doi: 10.3390/ijms22105246. PMID: 34063506 PMC8156342

[84] Yamamoto-Furusho JK Gutierrez-Herrera FD . Molecular mechanisms and clinical aspects of colitis-associated cancer in ulcerative colitis. Cells. (2025) 14:162. doi: 10.3390/cells14030162. PMID: 39936954 PMC11817687

[85] Mazzoleni A Awuah WA Sanker V Bharadwaj HR Aderinto N Tan JK . Chromosomal instability: a key driver in glioma pathogenesis and progression. Eur J Med Res. (2024) 29:451. doi: 10.1186/s40001-024-02043-8. PMID: 39227895 PMC11373396

[86] Kou F Wu L Ren X Yang L . Chromosome abnormalities: New insights into their clinical significance in cancer. Mol Ther Oncolytics. (2020) 17:562–70. doi: 10.1016/j.omto.2020.05.010. PMID: 32637574 PMC7321812

[87] Mio C Damante G . Challenges in promoter methylation analysis in the new era of translational oncology: a focus on liquid biopsy. Biochim Biophys Acta Mol Basis Dis. (2022) 1868:166390. doi: 10.1016/j.bbadis.2022.166390. PMID: 35296416

[88] Santacroce G Zammarchi I Nardone OM Capobianco I Puga-Tejada M Majumder S . Rediscovering histology – the application of artificial intelligence in inflammatory bowel disease histologic assessment. Ther Adv Gastroenterol. (2025) 18:17562848251325525. doi: 10.1177/17562848251325525. PMID: 40098604 PMC11912177

[89] Porcaro F Voccola S Cardinale G Porcaro P Vito P . DNA methylation biomarkers in stool samples: enhancing colorectal cancer screening strategies. Oncol Rev. (2024) 18:1408529. doi: 10.3389/or.2024.1408529. PMID: 39108328 PMC11300230

[90] Jiang Z Zhang H Gao Y Sun Y . Multi-omics strategies for biomarker discovery and application in personalized oncology. Mol BioMed. (2025) 6:115. doi: 10.1186/s43556-025-00340-0. PMID: 41269529 PMC12638490

[91] Kritzinger J Kotrri G Lakatos PL Bessissow T Wild G . The role of biomarkers in surveillance of ulcerative colitis-associated colorectal cancer: a scoping review. J Clin Med. (2025) 14:5979. doi: 10.3390/jcm14175979. PMID: 40943739 PMC12428994

[92] Nasreddin N Jansen M Loughrey MB Wang LM Koelzer VH Rodriguez-Justo M . Poor diagnostic reproducibility in the identification of nonconventional dysplasia in colitis impacts the application of histologic stratification tools. Mod Pathol. (2023) 37:100419. doi: 10.1016/j.modpat.2023.100419. PMID: 38158125

[93] Norton EJ Bateman AC . Pitfalls during histological assessment in locally resected pT1 colorectal cancer. Histopathology. (2025) 87:357–67. doi: 10.1111/his.15425. PMID: 39939288

[94] Lopez A Pouillon L Beaugerie L Danese S Peyrin-Biroulet L . Colorectal cancer prevention in patients with ulcerative colitis. Best Pract Res Clin Gastroenterol. (2018) 32–33:103–9. doi: 10.1016/j.bpg.2018.05.010. PMID: 30060933

[95] Lakhani AA Thompson SL Sheltzer JM . Aneuploidy in human cancer: new tools and perspectives. Trends Genet. (2023) 39:968–80. doi: 10.1016/j.tig.2023.09.002. PMID: 37778926 PMC10715718

[96] Hwang SH Baek SH Lee MJ Kook Y Bae SJ Ahn SG . Clinical relevance of TP53 mutation and its characteristics in breast cancer with long-term follow-up date. Cancers. (2024) 16:3899. doi: 10.3390/cancers16233899. PMID: 39682089 PMC11640694

[97] Endesfelder D Burrell RA Kanu N McGranahan N Howell M Parker PJ . Chromosomal instability selects gene copy-number variants encoding core regulators of proliferation in ER+ breast cancer. Cancer Res. (2014) 74:4853–63. doi: 10.1158/0008-5472.can-13-2664. PMID: 24970479 PMC4167338

[98] Milica K Filip M . Use of DNA methylation patterns for early detection and management of lung cancer: are we there yet? Oncol Res. (2024) 33:781–93. doi: 10.32604/or.2024.057231. PMID: 40191732 PMC11964873

[99] Yi Y . MicroRNA-mediated epigenetic regulation of inflammasomes in inflammatory responses and immunopathologies. Semin Cell Dev Biol. (2022) 154:227–38. doi: 10.1016/j.semcdb.2022.11.006. PMID: 36437174

[100] Yuan B Ma B Yu J Meng Q Du T Li H . Fecal bacteria as non-invasive biomarkers for colorectal adenocarcinoma. Front Oncol. (2021) 11:664321. doi: 10.3389/fonc.2021.664321. PMID: 34447694 PMC8383742

[101] East JE Gordon M Nigam GB Sinopoulou V Bateman AC Din S . British Society of Gastroenterology guidelines on colorectal surveillance in inflammatory bowel disease. Gut. (2025) 75:442–75. doi: 10.1136/gutjnl-2025-335023. PMID: 40306978 PMC13018778

[102] Iannone A Ruospo M Wong G Principi M Barone M Strippoli GF . Chromoendoscopy for surveillance in ulcerative colitis and Crohn’s disease: A systematic review of randomized trials. Clin Gastroenterol Hepatol. (2016) 15:1684–1697.e11. doi: 10.1016/j.cgh.2016.11.021. PMID: 27890853

[103] Lichtenstein GR Picco MF Solomon S Bickston SJ . The use of chromoendoscopy for surveillance of inflammatory bowel disease. VideoGIE. (2018) 3:35–42. doi: 10.1016/j.vgie.2017.11.004. PMID: 29905188 PMC5965708

[104] Pal P Singh AP Kanuri ND Banerjee R . Electronic chromo-endoscopy: technical details and a clinical perspective. Transl Gastroenterol Hepatol. (2021) 7:1–17. doi: 10.21037/tgh-19-373. PMID: 35243115 PMC8826039

[105] Hanen G Mohammed HE Nasser M Haseeb ME Yaser H Yaser S . Optimizing surveillance in Lynch syndrome: Lesion detection and comparative performance of different colonoscopy modalities—a systematic review and network meta-analysis. Int J Colorectal Dis. (2025) 40:175. doi: 10.1007/s00384-025-04970-2. PMID: 40796702 PMC12343729

[106] Maresca R Calabrese G Marchitto SA Schepis T Pecere S Maida M . Advanced diagnostic and resection endoscopic techniques in managing colitis-associated neoplasia: standard of care or still utopia? Best Pract Res Clin Gastroenterol. (2025) 78:102051. doi: 10.1016/j.bpg.2025.102051. PMID: 41350089

[107] Fasulo E D’Amico F Zilli A Furfaro F Cicerone C Parigi TL . Advancing colorectal cancer prevention in inflammatory bowel disease (IBD): Challenges and innovations in endoscopic surveillance. Cancers. (2024) 17:60. doi: 10.3390/cancers17010060. PMID: 39796690 PMC11718813

[108] Huguet JM Ferrer-Barceló L Suárez P Sanchez E Prieto JD Garcia V . Colorectal cancer screening and surveillance in patients with inflammatory bowel disease in 2021. World J Gastroenterol. (2022) 28:502–16. doi: 10.3748/wjg.v28.i5.502. PMID: 35316962 PMC8905018

[109] De Sire R De Deo D Mercurio M Franchellucci G Calabrese G Bonacci L . Sessile serrated lesions in inflammatory bowel disease: Hidden players in colitis-associated colorectal cancer? J Clin Med Res. (2025) 14:8042. doi: 10.3390/jcm14228042. PMID: 41303078 PMC12653639

[110] Bezzio C Della Corte C Vernero M Di Luna I Manes G Saibeni S . Inflammatory bowel disease and immune-mediated inflammatory diseases: looking at the less frequent associations. Ther Adv Gastroenterol. (2022) 15:1–16. doi: 10.1177/17562848221115312. PMID: 35924080 PMC9340394

[111] Smaje A Weston‐Clark M Raj R Orlu M Davis D Rawle M . Factors associated with medication adherence in older patients: A systematic review. Aging Med. (2018) 1:254–66. doi: 10.1002/agm2.12045. PMID: 31410389 PMC6692164

[112] Smith MJ Phillips RV Luque-Fernandez MA Maringe C . Application of targeted maximum likelihood estimation in public health and epidemiological studies: a systematic review. Ann Epidemiol. (2023) 86:34–48.e28. doi: 10.1016/j.annepidem.2023.06.004. PMID: 37343734

[113] Singh A Mahajan R Kedia S Dutta AK Anand A Bernstein CN . Use of thiopurines in inflammatory bowel disease: an update. Intest Res. (2021) 20:11–30. doi: 10.5217/ir.2020.00155. PMID: 33845546 PMC8831775

[114] Ranjan MK Kante B Vuyyuru SK Kumar P Mundhra SK Golla R . Minimal risk of lymphoma and non‐melanoma skin cancer despite long‐term use of thiopurines in patients with inflammatory bowel disease: a longitudinal cohort analysis from northern India. J Gastroenterol Hepatol. (2022) 37:1544–53. doi: 10.1111/jgh.15880. PMID: 35501287

[115] Limsrivilai J Lai AY Li STH Abdullah M Ali RR Aniwan S . Role of 5-aminosalicylic acid in ulcerative colitis management in 8 Asian territories: A physician survey. Intest Res. (2025) 23:117–28. doi: 10.5217/ir.2024.00085. PMID: 39757455 PMC12081079

[116] Adam L Phulukdaree A Soma P . Effective long-term solution to therapeutic remission in inflammatory bowel disease: Role of azathioprine. BioMed Pharmacother. (2018) 100:8–14. doi: 10.1016/j.biopha.2018.01.152. PMID: 29421584

[117] Cui G Fan Q Li Z Goll R Florholmen J . Evaluation of anti-TNF therapeutic response in patients with inflammatory bowel disease: Current and novel biomarkers. EBioMedicine. (2021) 66:103329. doi: 10.1016/j.ebiom.2021.103329. PMID: 33862588 PMC8054158

[118] McCormack MD Wahedna NA Aldulaimi D Hawker P . Emerging role of dual biologic therapy for the treatment of inflammatory bowel disease. World J Clin cases. (2023) 11:2621–30. doi: 10.12998/wjcc.v11.i12.2621. PMID: 37214562 PMC10198105

[119] Lakshmanan V Morris L . Beyond ursodeoxycholic acid: a comprehensive review of second-line agents in primary biliary cholangitis. Cureus. (2025) 17:e94172. doi: 10.7759/cureus.94172. PMID: 41209861 PMC12595517

[120] Sleiman J Kreidieh M Lee UJ Khouri P Plann-Curley B Sison C . Statins and the risk of colorectal cancer in patients with inflammatory bowel disease: a systematic review and meta-analysis. Gastroenterol Res. (2025) 18:108–18. doi: 10.14740/gr2028. PMID: 40503187 PMC12151120

[121] Seidel DV Azcárate-Peril MA Chapkin RS Turner ND . Shaping functional gut microbiota using dietary bioactives to reduce colon cancer risk. Semin Cancer Biol. (2017) 46:191–204. doi: 10.1016/j.semcancer.2017.06.009. PMID: 28676459 PMC5626600

[122] Zhang F Fan D Huang J Zuo T . The gut microbiome: linking dietary fiber to inflammatory diseases. Med Microecol. (2022) 14:100070. doi: 10.1016/j.medmic.2022.100070. PMID: 41878731

[123] Fiorucci S Urbani G Di Giorgio C Biagioli M Distrutti E . Bile acids-based therapies for primary sclerosing cholangitis: Current landscape and future developments. Cells. (2024) 13:1650. doi: 10.3390/cells13191650. PMID: 39404413 PMC11475195

[124] Koushki K Shahbaz SK Mashayekhi K Sadeghi M Zayeri ZD Taba MY . Anti-inflammatory action of statins in cardiovascular disease: The role of inflammasome and toll-like receptor pathways. Clin Rev Allergy Immunol. (2020) 60:175–99. doi: 10.1007/s12016-020-08791-9. PMID: 32378144 PMC7985098

[125] Bakhriansyah M Souverein PC De Boer A Klungel OH . Gastrointestinal toxicity among patients taking selective COX‐2 inhibitors or conventional NSAIDs, alone or combined with proton pump inhibitors: A case–control study. Pharmacoepidemiol Drug Saf. (2017) 26:1141–8. doi: 10.1002/pds.4183. PMID: 28370857 PMC5655916

[126] Ballester MP Marti-Aguado D Fullana M Bosca-Watts MM Tosca J Romero E . Impact and risk factors of non-adherence to 5-aminosalicylates in quiescent ulcerative colitis evaluated by an electronic management system. Int J Colorectal Dis. (2019) 34:1053–9. doi: 10.1007/s00384-019-03271-9. PMID: 30963247

[127] Tripathi K Dong J Mishkin BF Feuerstein JD . Patient preference and adherence to aminosalicylates for the treatment of ulcerative colitis. Clin Exp Gastroenterol. (2021) 14:343–51. doi: 10.2147/ceg.s237653. PMID: 34511961 PMC8412827

[128] Katona BW Weiss JM . Chemoprevention of colorectal cancer. Gastroenterology. (2019) 158:368–88. doi: 10.1053/j.gastro.2019.06.047. PMID: 31563626 PMC6981249

[129] Bezzio C Festa S Saibeni S Papi C . Chemoprevention of colorectal cancer in ulcerative colitis: Digging deep in current evidence. Expert Rev Gastroenterol Hepatol. (2017) 11:339–47. doi: 10.1080/17474124.2017.1292129. PMID: 28165825

